# The role of the comprehensive complication index for the prediction of survival after liver transplantation

**DOI:** 10.1007/s13304-020-00878-4

**Published:** 2020-09-06

**Authors:** Quirino Lai, Fabio Melandro, Greg Nowak, Daniele Nicolini, Samuele Iesari, Elisa Fasolo, Gianluca Mennini, Antonio Romano, Federico Mocchegiani, Kevin Ackenine, Marina Polacco, Laura Marinelli, Olga Ciccarelli, Giacomo Zanus, Marco Vivarelli, Umberto Cillo, Massimo Rossi, Bo-Göran Ericzon, Jan Lerut

**Affiliations:** 1 General Surgery and Organ Transplantation Unit, Department of Surgery, Sapienza University of Rome, Umberto I Polyclinic of Rome, Viale del Policlinico 155, 00161 Rome, Italy; 2 Division of Transplantation Surgery, Karolinska University Hospital Huddinge, Solna, Sweden; 3 Unit of Hepatobiliary Surgery and Transplantation, Polytechnic University of Marche, Azienda Ospedaliero-Universitaria “Ospedali Riuniti” Torrette, Ancona, Italy; 4 Starzl Unit of Abdominal Transplantation, Pôle de Chirurgie Expérimentale et Transplantation, Institut de Recherche Expérimentale et Clinique, Université Catholique de Louvain, Brussels, Belgium; 5 Department of Biotechnological and Applied Clinical Sciences, University of L’Aquila, L’Aquila, Italy; 6 Department of Surgery, Oncology and Gastroenterology, University of Padua, Padua, Italy

**Keywords:** MELD, Retransplantation, Graft survival, Survival prediction, Allograft dysfunction

## Abstract

In the last years, several scoring systems based on pre- and post-transplant parameters have been developed to predict early post-LT graft function. However, some of them showed poor diagnostic abilities. This study aims to evaluate the role of the comprehensive complication index (CCI) as a useful scoring system for accurately predicting 90-day and 1-year graft loss after liver transplantation. A training set (*n* = 1262) and a validation set (*n* = 520) were obtained. The study was registered at https://www.ClinicalTrials.gov (ID: NCT03723317). CCI exhibited the best diagnostic performance for 90 days in the training (AUC = 0.94; *p* < 0.001) and Validation Sets (AUC = 0.77; *p* < 0.001) when compared to the BAR, D-MELD, MELD, and EAD scores. The cut-off value of 47.3 (third quartile) showed a diagnostic odds ratio of 48.3 and 7.0 in the two sets, respectively. As for 1-year graft loss, CCI showed good performances in the training (AUC = 0.88; *p* < 0.001) and validation sets (AUC = 0.75; *p* < 0.001). The threshold of 47.3 showed a diagnostic odds ratio of 21.0 and 5.4 in the two sets, respectively. All the other tested scores always showed AUCs < 0.70 in both the sets. CCI showed a good stratification ability in terms of graft loss rates in both the sets (log-rank *p* < 0.001). In the patients exceeding the CCI ninth decile, 1-year graft survival rates were only 0.7% and 23.1% in training and validation sets, respectively. CCI shows a very good diagnostic power for 90-day and 1-year graft loss in different sets of patients, indicating better accuracy with respect to other pre- and post-LT scores.

Clinical Trial Notification: NCT03723317.

## Introduction

In the last years, several scoring systems have been developed with the intent to predict early clinical course after liver transplantation (LT). The model for end-stage liver disease (MELD) is recognized as the most accurate liver allograft allocation model, and it prioritizes patients according to the severity of their disease [1, 2]. However, several studies have shown that MELD alone fails to predict early post-transplant survival rates [3, 4]. Consequently, other scoring systems based on pre- or post-transplant available variables have been developed to identify cases with a high risk for transplant failure. Among them, the “pre-transplant” scores D-MELD and balance of risk (BAR) [5, 6], and the “post-transplant” score early allograft dysfunction (EAD) [7] proved to predict post-transplant survival satisfactorily.

Recently, the comprehensive complication index (CCI) has been developed to assess the actual complication rate after surgery [8]. Some reports have shown excellent prognostic power of this score in different fields [9–11]. No study to date has investigated the role of CCI in 90-day and 1-year prognostication of graft loss after LT.

This study aims to compare the abilities of CCI vs. other commonly adopted pre- and post-transplant scoring systems in diagnosing 90-day and 1-year post-transplant liver graft loss. The diagnostic capabilities were investigated in a training set and validated in a validation set.

## Materials and methods

Training set was generated retrospectively analyzing 1262 patients undergoing a first LT during the period January 1, 2005–December 31, 2016. Four European collaborative LT Centres were involved in creating the Training Set, namely the Polytechnic University of Marche, Ancona (Italy), Université Catholique de Louvain, Brussels (Belgium), Sapienza University, Rome (Italy), and University of Padua (Italy). Exclusion criteria for patient selection were: (a) living donation, (b) combined transplant, (c) domino transplant, and (d), pediatric (< 18 years) transplant.

Validation Set was created retrospectively analyzing the data of 520 patients transplanted during the same timeframe in the Karolinska Institute of Stockholm (Sweden). The same exclusion criteria were adopted. The study was registered at https://www.ClinicalTrials.gov (ID: NCT03723317).

### Definitions

Organ procurement was defined as “local” when done in the same region in which the LT was performed. All complications were graded according to the Clavien–Dindo Classification [12]. A web-calculator was used for estimating BAR and CCI (available at https://www.assessurgery.com/).

The CCI was calculated using the following original algorithm: CCI = [√(wC1 + wC2 … + wC*x*)]/2.

The CCI is based on the complication grading by Clavien–Dindo Classification and implements every occurred weighted complication (wC) after an intervention. Clavien–Dindo grade I corresponds to 8.7, grade II to 20.9, grade IIIa to 26.2, grade IIIb to 33.7, grade IVa to 42.4, grade IVb to 46.2, and grade V to 100. All the complications collected were summed, even if the same patient received several times multiple administrations of the same medical (i.e., blood transfusion) or interventional (i.e., various radiological or surgical approaches) treatment. The overall morbidity is reflected on a scale from 0 (no complication) to 100 (death).

Retransplantation during the first hospitalization was calculated as IVa (liver failure) plus IIIb (reoperation) complication. Multiorgan failure (MOF) was defined as the presence of at least two organ failures and ranked as grade IVb complication. Primary non-function (PNF) was identified as a liver failure observed for non-technical reasons within seven days after surgery and ranked as IVa complication.

EAD was defined according to the Olthoff criteria [7], and classified as grade II complication. Mild renal dysfunction was associated with a serum creatinine increase overpassing the threshold of 1.5 mg/dL but not requiring renal replacement therapy (RRT), and corresponded to a grade I complication. In the case of RRT, a grade IVa complication was defined. Myelotoxicity was defined as the presence of at least one of the following conditions: anemia (hemoglobin < 8 g/dL) in the absence of bleeding, leukopenia (< 3500/μL), or severe thrombocytopenia (< 30,000/μL), being classified as a grade I complication.

### Statistical analysis

Continuous variables were reported as medians and interquartile ranges (IQR). Categorical variables were reported as numbers and percentages. Missing data always involved < 10% per variable and were handled using the maximum likelihood estimation method. Mann–Whitney *U* test was used for comparisons between groups in case of continuous variables, and Fisher’s exact test was adopted in case of categorical variables.

A univariate Cox regression analysis was performed in the training set for the identification of the risk factors for graft loss. All the variables with a *p* value < 0.20 were introduced into a multivariable model. A multivariable Cox regression model was constructed adopting the backward conditional method [13]. Beta-coefficients, standard errors, the hazard ratio (HR), and 95% confidence intervals (95% CI) were reported.

C-statistics was used for comparing the diagnostic ability of different scores in terms of 90-day and 1-year graft loss in both the training and validation set. Specifically, CCI was compared with MELD, D-MELD, BAR, and EAD. Areas under the curve (AUC), standard errors, and 95% CI were reported. The following CCI cut-off values were investigated in the training set: first quartile, median, third quartile, and ninth decile. Sensitivity, specificity, and diagnostic odds ratio (DOR) were reported for each cut-off value. The higher the DOR value, the greater its discriminative power. The same CCI threshold values obtained in the training set were validated in the Validation Set. Graft survival rates were estimated with the Kaplan–Meier method; the log-rank test was used for evaluating survival differences. A *p* value < 0.05 was considered statistically significant in all analyses. Statistical reports and plots were performed using the SPSS statistical package version 24.0 (SPSS Inc., Chicago, IL, USA).

## Results

The training and validation sets were composed of 1262 and 520 LT recipients. All grafts were procured from donors after brain death.

In the training set, the median follow-up was 3.7 years (IQR 1.1–7.6), with 1108 (87.8%) and 991 (78.5%) cases exceeding 90 days and 1 year, respectively.

During the entire study period, 371 (29.4%) patients died: of whom 154 (12.8%) and 66 (5.2%) within 90 days and during the time interval of 91–365 days, respectively.

Four hundred and nine (32.4%) grafts were lost: 186 (14.7%) and 70 (5.5%) within 90 days and 91–365 days, respectively. Seventy-eight (6.2%) retransplantations were performed: 54 (4.5%) and 12 (1.0%) within 90 days and 91–365 days, respectively.

In the validation set, the median follow-up was 4.8 years (IQR 3.0–7.2), with 504 (97.9%) and 485 (93.3%) cases exceeding 90 days and 1 year, respectively.

During the entire study period, 104 (20.0%) patients died: 15 (2.9%) within 90 days and 18 (3.5%) during the time interval of 91–365 days from LT.

One hundred and fourteen (21.9%) grafts were lost: 17 (3.3%) within 90 days and 20 (3.8%) during 91–365 days. Twelve (2.3%) retransplantations were performed: one (0.2%) within 90 days and three (0.6%) during 91–365 days.

### Baseline characteristics

The characteristics of the sets are displayed in Table 1.

**Table 1:** Characteristics of recipients, donors, and transplants in the training and validation sets

Variables	Median (IQR) or *n* (%)		*p* value
	Training set (*N* = 1262)	Validation set (*N* = 520)	
Recipient			
Age at LT (years)	56 (49–62)	54 (44–62)	0.001
Male gender	949 (75.2)	355 (68.3)	0.003
Waiting time (months)	4 (1–10)	2 (1–5)	0.003
MELD la	15 (10–22)	26 (23–29)	< 0.001
Disease			
HCC	527 (41.8)	131 (25.2)	< 0.001
HCV	451 (35.7)	148 (28.5)	0.003
HBV	174 (13.8)	35 (6.7)	< 0.001
Alcohol	433 (34.3)	109 (21.0)	< 0.001
Acute liver failure	64 (5.1)	20 (3.8)	0.3
NASH	88 (7.0)	47 (9.0)	0.1
Other	223 (17.7)	235 (45.2)	< 0.001
Donor			
Age (years)	57 (43–70)	57 (44–67)	0.2
Male gender	719 (57.0)	292 (56.2)	0.8
BMI (kg/m^2^)	25 (23–28)	25 (22–28)	0.06
ICU stay (days)	3 (2–5)	2 (1–3)	< 0.001
Cause of death			
Trauma	337 (26.7)	76 (14.6)	< 0.001
Anoxia	94 (7.4)	92 (17.7)	< 0.001
CVA	785 (62.2)	346 (66.5)	0.09
Other	62 (4.9)	5 (1.0)	< 0.001
Cardiac arrest	158 (12.5)	133 (25.6)	< 0.001
History of DM	90 (7.1)	39 (7.5)	0.8
Local procurement	640 (50.7)	387 (74.4)	< 0.001
Transplant			
Cold ischemia time (min) Warm ischemia time (min) D-MELD BAR	434 (360–533) 45 (35–60) 815 (499–1245) 5 (3–9)	496 (411–568) 40 (34–50) 1454 (1084–1826) 11 (8–13)	< 0.001 < 0.001 < 0.001 < 0.001
Post-LT clinical course			
ICU stay (days)	4 (2–7)	1 (1–2)	< 0.001
Total length of stay (days) Total bilirubin seventh day (mg/dL) INR 7th day ALT peak during first week (IU/L) AST peak during first week (IU/L)	17 (13–27) 4.5 (1.8–8.3) 1.00 (1.00–1.16) 745 (382–1509) 907 (447–2086)	14 (11–20) 2.1 (1.0–4.2) 1.20 (1.10–1.40) 364 (202–707) 252 (143–447)	< 0.001 < 0.001 < 0.001 < 0.001 < 0.001

*LT* liver transplantation, *IQR* interquartile ranges, *MELD* model for end-stage liver disease, *HCC* hepatocellular cancer, *HCV* hepatitis C virus, *HBV* hepatitis B virus, *NASH* non-alcoholic steatohepatitis, *BMI* body mass index, *ICU* intensive care unit, *CVA* cerebrovascular accident, *DM* diabetes mellitus, *D-MELD* donor-MELD, *BAR* balance of risk, *INR* international normalized ratio, *ALT* alanine aminotransferase, *AST* aspartate aminotransferase

In the training set, median lab-MELD was 15 points, with 161 (12.8%) patients showing a MELD ≥ 30. Median waiting time and age at LT were 4 months and 56 years, respectively. HCC was the main indication for LT in 527 (41.8%) patients. The main cause of the liver disease was HCV-related cirrhosis (35.7%). Median donor age was 57 years, with 328 (26.0%) and 77 (6.1%) donors exceeding 70 and 80 years. The leading brain-death cause was cerebrovascular accident (*n* = 785; 62.2%). In approximately half of the cases, the procurement was performed in a local hospital. Median cold and warm ischemia times were 7.2 h and 45 min. Median BAR score was 5; 77 (6.1%) and six (0.5%) recipients had a score exceeding 15 and 20, respectively.

In the validation set, median lab-MELD was 25 points, with 118 (22.7%) recipients presenting a MELD ≥ 30. Median waiting time and age at LT were 2 months and 54 years, respectively. HCC was the leading indication for LT in 131 (25.2%) patients. HCV-related cirrhosis was reported in 148 (28.5%) cases. Pathologies uncommonly reported in the Training Set were, on the opposite, commonly reported in the validation set: biliary pathologies like primary biliary cholangitis and primary sclerosing cholangitis were reported in 124/520 (23.8%) cases, followed by 37 (7.1%) cases of familiar amyloid polyneuropathy, and 25 (4.8%) cases of autoimmune hepatitis.

Median donor age was 57 years, with 91 (17.5%) and eight (1.5%) donors exceeding 70 and 80 years. The leading cause of brain death was a cerebrovascular accident (*n* = 346; 66.5%). In ~ 75% of cases, the procurement was performed in a local hospital. Median cold and warm ischemia times were 8.3 h and 40 min. Median BAR score was 11; 30 (5.8%) recipients had a score exceeding 15, while no case exceed 20.

### Post-transplant course

The postoperative courses of the two sets are displayed in Table 2. In the training set, median intensive care stay and overall length of hospital stay were four and 17 days. According to the highest Clavien–Dindo grade, 182 (14.4%) patients had no complications, 184 (14.6%) and 406 (32.2%) had grades I and II, 79 (6.2%) and 146 (11.5%) had grades IIIa and IIIb, 96 (7.7%) and 21 (1.7%) had grades IVa and IVb, and 148 (11.7%) died (grade V). One hundred and fifty-eight (12.5%) patients required a “IIIa procedure”. The most common procedures were thoracic (*n* = 54; 4.3%) or abdominal drainage (*n* = 45; 3.6%). Two hundred and seventy-one (21.5%) patients required a “IIIb procedure”. The most common procedure was reoperation for bleeding (*n* = 88; 7.0%), followed by explorative laparotomy (*n* = 73; 5.8%). Retransplantation during the same hospital stay of the first transplant was necessary for 45 (3.6%) recipients. MOF was observed in 45 (3.6%) recipients. EAD and PNF were reported in 508 (40.3%) and 37 (2.9%) cases. Mild renal dysfunction and RRT were detected in 90 (7.1%) and 91 (7.2%) patients. Vascular and biliary complications were reported in 80 (6.3%) and 110 (8.7%) subjects. Median CCI value was 29.3 (IQR: 12.2–47.6): CCI values < 20, 20–39, 40–49, 50–99, and 100 were observed in 366 (29.0%), 483 (38.3%), 121 (9.6%), 144 (11.4%), and 148 (11.7%) patients.

**Table 2:** Interventional procedures performed and complications reported in the training and validation sets

Variables	Training set (*N* = 1262)	Validation set (*N* = 520)	*p* value
	Median (IQR) and *n* (%)		
CD score IIIa	158 (12.5)	134 (25.8)	< 0.001
Biliary stenting	34 (2.7)	29 (5.6)	0.004
HA stenting	10 (0.8)	1 (0.2)	0.2
Abdominal drainage	45 (3.6)	38 (7.3)	0.001
Thoracic drainage	54 (4.3)	55 (10.6)	< 0.001
Arterial embolization	17 (1.3)	0 (–)	0.005
Other	28 (2.2)	42 (8.1)	< 0.001
CD score IIIb	271 (21.5)	101 (19.4)	0.4
Bleeding control	88 (7.0)	41 (7.9)	0.5
Immediate retransplantation	45 (3.6)	3 (0.6)	< 0.001
Biliary redo	61 (4.8)	9 (1.7)	0.002
HA/PV redo	36 (2.9)	2 (0.4)	< 0.001
Explorative laparotomy	73 (5.8)	33 (6.3)	0.7
Tracheotomy	20 (1.6)	11 (2.1)	0.4
Depacking	14 (1.1)	1 (0.2)	0.08
Two-time closure	6 (0.5)	5 (1.0)	0.3
Other	27 (2.1)	26 (5.0)	< 0.001
MOF	45 (3.6)	12 (2.3)	0.2
PNF	37 (2.9)	3 (0.6)	0.001
Cardiac failure/ischemic	32 (2.5)	10 (1.9)	0.5
HD replacement	91 (7.2)	39 (7.5)	0.8
Respiratory failure	39 (3.1)	31 (6.0)	0.007
EAD	508 (40.3)	87 (16.7)	< 0.001
Acute rejection	211 (16.7)	95 (18.3)	0.4
HAT	38 (3.0)	1 (0.2)	< 0.001
HA stenosis	22 (1.7)	0 (-)	0.001
PV thrombosis/stenosis	20 (1.6)	1 (0.2)	0.01
Biliary stenosis	48 (3.8)	10 (1.9)	0.04
Biliary fistula	62 (4.9)	1 (0.2)	< 0.001
Abdominal bleeding	108 (8.6)	3 (0.6)	< 0.001
Infection	438 (34.7)	188 (36.2)	0.6
Neurological/psychiatric	202 (16.0)	98 (18.8)	0.2
Cardiac electric	32 (2.5)	29 (5.6)	0.002
Mild acute renal dysfunction	90 (7.1)	19 (3.7)	0.005
Ascites	225 (17.8)	49 (9.4)	< 0.001
Diarrhoea	23 (1.8)	25 (4.8)	0.001
Gastrointestinal bleeding	21 (1.7)	28 (5.4)	< 0.001
Myelotoxicity	149 (11.8)	40 (7.7)	0.01
Intestinal occlusion/peritonitis	21 (1.7)	3 (0.6)	0.07
Pneumothorax	11 (0.9)	1 (0.2)	0.2
CCI	29.3 (12.2–47.6)	24.2 (8.7–44.4)	0.3

*IQR* interquartile ranges, *CD* Clavien–Dindo, *HA* hepatic artery, *LT* liver transplantation, *PV* portal vein, *MOF* multiorgan failure, *PNF* primary non-function, *HD* hemodialysis, *EAD* early allograft dysfunction, *HAT* hepatic artery thrombosis, *CCI* comprehensive complication index

In the validation set, median intensive care stay and overall length of hospital stay were one and 14 days. According to the highest Clavien–Dindo grade observed, 148 (28.5%) and 152 (29.2%) patients had a grade I and II, 77 (14.8%) and 75 (14.4%) had grades IIIa and IIIb, 46 (8.8%) and 13 (2.5%) had grades IVa and IVb, and, lastly, nine (1.7%) had a grade V complication. A total of 134 (25.8%) patients required a “IIIa procedure”. The most common procedures were thoracic (*n* = 55; 10.6%) or abdominal drainage (*n* = 38; 7.3%). Two hundred and one (19.4%) patients required a “IIIb procedure”. The most common was reoperation for bleeding (*n* = 41; 7.9%), followed by explorative laparotomy (*n* = 33; 6.3%). Early retransplantation was necessary in only three (0.6%) cases. MOF was diagnosed in 12 (2.3%) recipients. EAD and PNF were observed in 87 (16.7%) and three (0.6%) cases. Mild renal dysfunction and RRT were reported in 19 (3.7%) and 39 (7.5%) patients. Vascular and biliary complications were reported in two (0.4%) and 11 (2.1%) subjects. Median CCI value was 24.2 (IQR: 8.7–44.4): CCI values < 20, 20–39, 40–49, 50–99, and 100 were observed in 148 (28.5%), 220 (42.3%), 57 (11.0%), 86 (16.5%), and nine (1.7) patients.

### Risk factors for overall risk of graft loss

Eighteen different covariates identifiable before or during the post-LT hospital stay were tested in the training set. First, a univariate Cox regression analysis was displayed with the intent to identify the risk factors for graft loss. After selecting only the statistically significant variables, and removing the possible causes of co-linearity, a multivariable Cox regression model was built. Three independent risk factors for graft loss were identified: donor age (HR = 1.01; *p *value = 0.002), BAR score (HR = 1.03; *p *value = 0.01) and CCI (HR = 1.05; *p *value < 0.001) (Table 3). Interestingly, CCI presented a very high Wald value (552.95) with respect to donor age and BAR (9.38 and 6.58, respectively), thus showing a high contribution of this individual predictor in the construction of the given model.

**Table 3:** Univariable and multivariable Cox regression analyses for the overall risk of graft loss after LT in the training set

Variables	Training set (*N* = 1262)								
	Univariable analysis				Multivariable analysis				
	Beta-coefficient ± SE	OR	95% CI	*p* value	Beta-coefficient ± SE	Wald	HR	95% CI	*p* value
Waiting time (per day)	0.00 ± 0.00	1.00	1.00–1.00	0.7	–	–	–	–	–
Patient age at LT (per year)	− 0.00 ± 0.01	1.00	0.99–1.01	0.8	–	–	–	–	–
Patient male gender (yes vs. no)	0.11 ± 0.12	1.11	0.89–1.40	0.4	–	–	–	–	–
Patient BMI at LT (per unit)^a^	− 0.02 ± 0.01	0.98	0.96–1.00	0.08	–	–	–	–	–
MELD at LT (per point)^b^	0.02 ± 0.01	1.02	1.01–1.03	0.002	–	–	–	–	–
Donor age (per year)^a^	0.01 ± 0.00	1.01	1.00–1.02	0.001	0.01 ± 0.00	9.38	1.01	1.00–1.01	0.002
Donor male gender (yes vs. no)	− 0.06 ± 0.10	0.94	0.77–1.14	0.5	–	–	–	–	–
Donor BMI at LT (per unit)	0.01 ± 0.01	1.01	0.98–1.03	0.5	–	–	–	–	–
CVA as cause of donor death^a^	0.26 ± 0.10	1.29	1.05–1.58	0.01	–	–	–	–	–
Donor ICU stay (per day)	0.00 ± 0.01	1.00	0.98–1.03	0.9	–	–	–	–	–
Donor DM2^a^	0.26 ± 0.18	1.30	0.91–1.85	0.2	–	–	–	–	–
Donor local procurement	− 0.09 ± 0.10	0.91	0.75–1.11	0.4	–	–	–	–	–
CIT (per min)	0.00 ± 0.00	1.00	1.00–1.00	0.5	–	–	–	–	–
WIT (per min)^a^	0.01 ± 0.00	1.01	1.00–1.01	0.003	–	–	–	–	–
BAR score (per point)^a^	0.04 ± 0.01	1.04	1.01–1.06	0.002	0.03 ± 0.01	6.58	1.03	1.01–1.05	0.01
D-MELD (per 100 points)^a,b^	0.03 ± 0.01	1.03	1.02–1.05	< 0.001	–	–	–	–	–
EAD^a^	0.44 ± 0.10	1.55	1.28–1.89	< 0.001	–	–	–	–	–
CCI^a^	0.04 ± 0.00	1.04	1.04–1.05	< 0.001	0.04 ± 0.00	552.95	1.05	1.04–1.05	< 0.001

The Backward Wald method was used for selecting the covariates in the multivariable analyses. − 2log likelihood = 4,955.53

*SE* standard error, *HR* hazard ratio, *CI* confidence intervals, *LT* liver transplantation, *BMI* body mass index, *MELD* model for end-stage liver disease, *CVA* cerebrovascular accident, *ICU* intensive care unit, *DM* diabetes mellitus, *CIT* cold ischemia time, *WIT* warm ischemia time, *BAR* balance of risk, *D-MELD* donor-MELD, *EAD* early allograft dysfunction, *CCI* comprehensive complication index

^a^Variables initially introduced in the multivariable model

^b^The multivariable model was constructed introducing only the variable “D-MELD”, with the intent to avoid collinearity phenomena with the variable “MELD”. A similar model with the variable “MELD” instead of “D-MELD” was contextually constructed. In both cases, D-MELD or MELD were deleted during the backward Wald method

### 90-Day graft loss diagnostic ability

The diagnostic ability of five different scoring systems was evaluated in both the sets, with the intent to identify the best diagnostic test for 90-day graft loss (Table 4).

**Table 4:** Prediction of 90-day graft loss in the training and validation sets

Scores	Training set (*N* = 1262)			Scores	Validation set (*N* = 520)		
	AUC ± SE	95% CIs	*p* value		AUC ± SE	95% CIs	*p* value
CCI	0.94 ± 0.01	0.92–0.96	< 0.001	CCI	0.77 ± 0.08	0.62–0.93	< 0.001
D-MELD	0.60 ± 0.02	0.56–0.65	< 0.001	BAR	0.57 ± 0.06	0.45–0.68	0.36
MELD	0.60 ± 0.02	0.56–0.65	< 0.001	EAD	0.57 ± 0.07	0.43–0.71	0.35
BAR	0.60 ± 0.02	0.55–0.64	< 0.001	D-MELD	0.56 ± 0.08	0.41–0.70	0.43
EAD	0.58 ± 0.02	0.53–0.62	0.001	MELD	0.47 ± 0.07	0.33–0.61	0.70

CCI cut-off	Sens	Spec	DOR	CCI cut-off	Sens	Spec	DOR
12.2 (25th)	98.4	27.7	23.6	12.2	82.4	32.1	2.2
29.6 (50th)	96.8	56.1	38.7	29.6	76.5	52.1	3.5
47.3 (75th)	88.8	85.9	48.3	47.3	64.7	79.3	7.0
84.9 (90th)	66.3	98.7	149.4	84.9	41.2	98.6	49.3

*AUC* area under the curve, *SE* standard error, *CIs* confidence intervals, *CCI* comprehensive complication index, *D-MELD* donor-model for end-stage liver disease, *MELD* model for end-stage liver disease, *EAD* early allograft dysfunction, *BAR* balance of risk, *DOR* diagnostic odds ratio

CCI exhibited the best diagnostic performances in both Training (AUC = 0.94, 95% CI = 0.92–0.96; *p* < 0.001) and Validation Sets (AUC = 0.77, 95% CI = 0.62–0.93; *p* < 0.001) when compared to the BAR, D-MELD, MELD, and EAD scores. All the other scores always showed inferior AUCs, only ranging 0.58–0.60 and 0.47–0.57, respectively.

In the training set, the CCI cut-off value corresponding to the first quartile (12.2 points) yielded a sensitivity of 98.4 and a specificity of 27.7 (DOR = 23.6). The threshold value corresponding to the median point (29.6) had a sensitivity of 96.8 and a specificity of 56.1 (DOR = 38.7). The value of 47.3, corresponding to the third quartile, exhibited a sensitivity of 88.8 and a specificity of 85.9, giving a high DOR value of 48.3. Lastly, the threshold value put at 84.9 (ninth decile) showed a sensitivity = 66.3 and a specificity = 98.7 (DOR = 149.4) (Table 4).

The same cut-offs validated in the validation set showed similar excellent diagnostic ability, although they were inferior in terms of discriminative power.

The CCI cut-off value at 12.2 (first quartile) yielded a sensitivity of 82.4 and a specificity of 32.1 (DOR = 2.2). The cut-off set at 29.6 (median) had a sensitivity of 76.5 and a specificity of 52.1 (DOR = 3.5). The cut-off put at 47.3 (third quartile) exhibited a sensitivity of 64.7 and a specificity of 79.3, giving a DOR value of 7.0. Lastly, the threshold value put at 84.9 (ninth decile) presented a sensitivity = 41.3 and a specificity = 98.6 (DOR = 49.3) (Table 4).

### 1-year graft loss diagnostic ability

The diagnostic ability of five different scoring systems was evaluated in both the sets, with the intent to identify the best diagnostic test for 1-year graft loss (Table 5).

**Table 5:** Prediction of 1-year graft loss in the training and validation sets

Scores	Training set (*N* = 1262)			Scores	Validation set (*N* = 520)		
	AUC ± SE	95% CIs	*p* value		AUC ± SE	95% CIs	*p* value
CCI	0.88 ± 0.02	0.85–0.90	< 0.001	CCI	0.75 ± 0.05	0.65–0.85	< 0.001
D-MELD	0.59 ± 0.02	0.55–0.63	< 0.001	D-MELD	0.63 ± 0.05	0.54–0.73	0.007
MELD	0.59 ± 0.02	0.55–0.63	< 0.001	MELD	0.54 ± 0.05	0.45–0.63	0.40
BAR	0.59 ± 0.02	0.55–0.63	< 0.001	EAD	0.53 ± 0.05	0.43–0.63	0.50
EAD	0.56 ± 0.02	0.52–0.60	0.004	BAR	0.45 ± 0.05	0.36–0.55	0.32

CCI cut-off	Sens	Spec	DOR	CCI cut-off	Sens	Spec	DOR
12.2 (25th)	94.9	28.6	7.5	12.2	86.5	33.1	3.2
29.6 (50th)	89.1	57.8	11.2	29.6	78.4	53.4	4.2
47.3 (75th)	75.0	87.5	21.0	47.3	56.8	80.5	5.4
84.9 (90th)	53.5	99.9	1,149.4	84.9	27.0	99.2	45.9

*AUC* area under the curve, *SE* standard error, *CIs* confidence intervals, *CCI* comprehensive complication index, *D-MELD* donor-model for end-stage liver disease, *MELD* model for end-stage liver disease, *EAD* early allograft dysfunction, *BAR* balance of risk, *DOR* diagnostic odds ratio

CCI exhibited the best diagnostic performances in both training (AUC = 0.88, 95% CI = 0.85–0.90; *p* < 0.001) and validation sets (AUC = 0.75, 95% CI = 0.65–0.85; *p* < 0.001). All the other scores always showed inferior AUCs, only ranging 0.56–0.59 and 0.45–0.63, respectively.

In the training set, the CCI cut-off value corresponding to the first quartile (12.2 points) yielded a sensitivity of 94.9 and a specificity of 28.6 (DOR = 7.5). The threshold value corresponding to the median point (29.6) had a sensitivity of 89.1 and a specificity of 57.8 (DOR = 11.2). The value of 47.3, corresponding to the third quartile, exhibited a sensitivity of 75.0 and a specificity of 87.5, giving a high DOR value of 21.0. Lastly, the threshold value put at 84.9 (ninth decile) showed a sensitivity = 53.5 and a specificity = 99.9 (DOR = 1149.4) (Table 5).

The same cut-offs validated in the validation set showed similar excellent diagnostic ability, although they were inferior in terms of discriminative power.

The CCI cut-off value at 12.2 (first quartile) yielded a sensitivity of 86.5 and a specificity of 33.1 (DOR = 3.2). The cut-off set at 29.6 (median) had a sensitivity of 78.4 and a specificity of 53.4 (DOR = 4.2). The cut-off put at 47.3 (third quartile) exhibited a sensitivity of 56.8 and a specificity of 80.5, giving a DOR value of 5.4. Lastly, the threshold value put at 84.9 (ninth decile) presented a sensitivity = 27.0 and a specificity = 99.2 (DOR = 45.9) (Table 5).

### Sub-analysis on the graft loss diagnostic ability using aged grafts

The diagnostic ability of the five different scoring systems was also evaluated in a sub-analysis in which only transplants performed using organs from aged (≥ 70 years) donors were considered (Table 6). As for the 90-day risk of graft loss, CCI confirmed the best diagnostic performances in both training (AUC = 0.93, 95% CI = 0.88–0.97; *p* < 0.001) and validation sets (AUC = 0.92, 95% CI = 0.81–1.00; *p* = 0.001). Similarly, CCI was also the best diagnostic tool for predicting 1-year graft loss, with the best diagnostic performances in both training (AUC = 0.88, 95% CI = 0.82–0.93; *p* < 0.001) and validation sets (AUC = 0.79, 95% CI = 0.59–1.00; *p* = 0.002).

**Table 6:** Prediction of 90-day and 1-year graft loss in the training and validation sets: transplants performed using organs from donors with age ≥ 70 years

Scores	Training set (*N* = 1262)			Scores	Validation set (*N* = 520)		
	AUC ± SE	95% CIs	*p* value		AUC ± SE	95% CIs	*p* value
90-Day graft loss							
CCI	0.93 ± 0.02	0.88–0.97	< 0.001	CCI	0.92 ± 0.06	0.81–1.00	0.001
BAR	0.59 ± 0.04	0.52–0.67	0.03	BAR	0.55 ± 0.11	0.34–0.76	0.69
EAD	0.53 ± 0.04	0.45–0.61	0.50	D-MELD	0.53 ± 0.12	0.30–0.77	0.80
MELD	0.53 ± 0.05	0.44–0.61	0.52	EAD	0.50 ± 0.12	0.26–0.74	0.99
D-MELD	0.52 ± 0.05	0.43–0.61	0.64	MELD	0.46 ± 0.11	0.26–0.68	0.77
1-year graft loss							
CCI	0.88 ± 0.03	0.82–0.93	< 0.001	CCI	0.79 ± 0.10	0.59–1.00	0.002
BAR	0.57 ± 0.04	0.50–0.64	0.06	BAR	0.53 ± 0.10	0.34–0.71	0.78
EAD	0.53 ± 0.04	0.46–0.60	0.49	EAD	0.52 ± 0.09	0.33–0.70	0.87
D-MELD	0.52 ± 0.04	0.44–0.59	0.69	D-MELD	0.49 ± 0.11	0.28–0.70	0.95
MELD	0.52 ± 0.04	0.45–0.60	0.51	MELD	0.47 ± 0.10	0.27–0.66	0.71

*AUC* area under the curve, *SE* standard error, *CIs* confidence intervals, *CCI* comprehensive complication index, *D-MELD* donor-model for end-stage liver disease, *MELD* model for end-stage liver disease, *EAD* early allograft dysfunction, *BAR* balance of risk, *DOR* diagnostic odds ratio

### Graft survival rates

In the training set, we obtained an excellent stratification of graft survival rates using the investigated CCI thresholds.

**Fig. 1:**
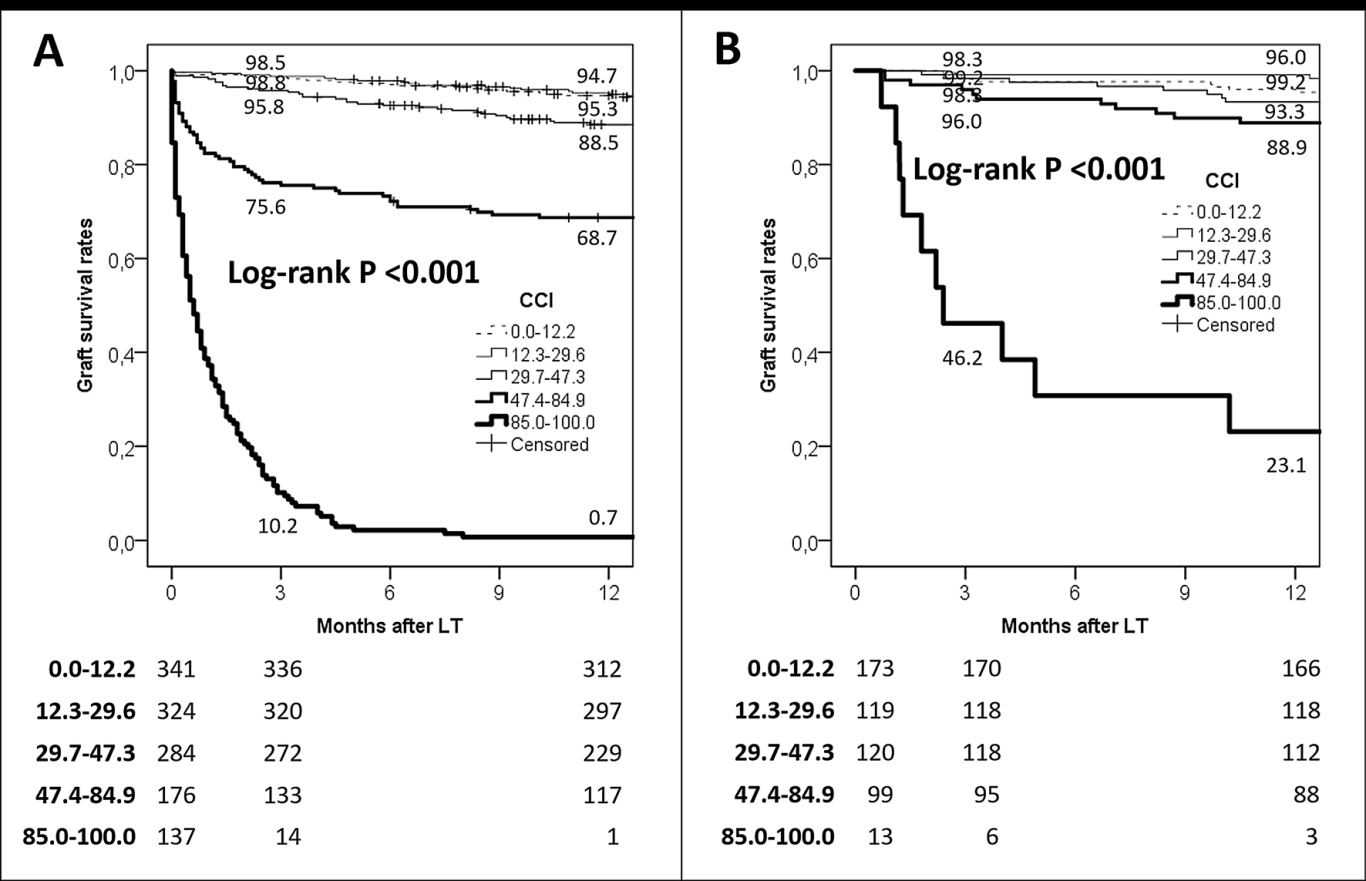
**a** Training set: 1-year graft survival rates according to the CCI risk strata. **b** Validation set: 1-year graft survival rates according to the CCI risk strata

For instance, 1-year graft survival rates were 94.7, 95.3, 88.5, 68.7, and 0.7% in patients with CCI 0.0–12.2, 12.3–29.6, 29.7–47.3, 47.4–84.9, and 85.0–100.0, respectively (log-rank *p* < 0.001) (Fig. 1a).

In the validation set, we similarly obtained a good stratification of graft survivals. For instance, 1-year graft survival rates were 96.0, 99.2, 93.3, 88.9, and 23.1% in patients with CCI 0.0–12.2, 12.3–29.6, 29.7–47.3, 47.4–84.9, and 85.0–100.0, respectively (log-rank *p* < 0.001) (Fig. 1b).

## Discussion

A valid scoring system should exhibit good performance metrics, such as discrimination and calibration, to maintain these qualities over time, and it should be simple and easy to calculate.

In the specific setting of LT, MELD score covers most of these characteristics when used for the prediction of death during the waiting time. This ability was the reason for the introduction of the MELD score, in 2002, in the US liver allocation process. The goal was to prioritize the sickest patients for transplantation [14]. However, the MELD score rapidly proved to be a poor predictor of short- and, worse, long-term post-transplant survival [15–17]. A recent systematic review, which included 37 studies covering 53,691 patients transplanted in 15 different countries, identified an overall c-statistics inferior to 0.7 and consequently suggested a global poor predictive value of the score [4].

With the intent to improve its predictive ability, the MELD score has been integrated into different models built to enhance the prediction of post-transplant survival. Unfortunately, the complexity of many of these models limits their usability. The Survival Outcomes Following Liver Transplantation (SOFT) score represents a paradigmatic example: despite its good predictive ability, the score is based on 18 different pre-transplant variables, making it difficult to calculate [18]. The same holds for the MELD-sarcopenia score, in which the single complex-to-estimate parameter “sarcopenia” limits its broad applicability [19]. Conversely, the D-MELD, based on the simple multiplication of donor age and recipient MELD, represents an easy-to-calculate model [5, 20]. The BAR score, based on six donor- and recipient-related pre-operative variables, further improves prognostication without excessively increasing the complexity [6]. Moreover, a web calculator is available for its estimation. The BAR score has proven to offer great potential in different geographical areas [6, 21, 22]. A Chinese study including 249 LDLT patients showed that the BAR score was the best predictor of 1-year patient survival [21]. A Brazilian study including 402 patients reported similar results when looking at three-month patient survival [22].

However, all these scores based on pre-transplant data typically yielded inferior results compared to scoring systems based on variables available in the immediate post-transplant period. Among the post-transplant scores, the Olthoff-EAD is the most commonly adopted [7, 23].

The great and largely unsolved challenge in LT remains how to correctly allocate a limited resource such as organs from deceased donors, which can be addressed only with preoperative variables. Therefore, a score composed by post-transplant parameters cannot be used with the intent to optimize the allocation process. However, such a score should maintain its potential usefulness as a diagnostic tool for early (i.e., 3-month, 1-year) clinical course prediction.

In the present series, we observed that the CCI model presented high relevance for LT survival prognostication in both the Sets we investigated. Moreover, CCI outperformed both the pre- and post-transplant scores in diagnostic ability.

CCI was initially created to report complication rates more accurately. The CCI aimed to inform about the severity of cumulative postoperative complications precisely [8]. Recently, its potential role as a prognostic tool has been implemented in different fields of surgery. As an example, two international studies used CCI cut-off values of 33 and 42 as benchmarks for evaluating the quality of a successfully performed liver resection or transplantation, respectively [10, 24].

Several studies investigated the prognostic impact of CCI in the setting of different types of cancer. A US study showed that CCI was a strong survival predictor in patients undergoing hepatic resection for colorectal metastases independently from the RAS mutational status. Patients with high CCI (≥ 26.2) had worse recurrence-free and cancer-specific survivals with respect to low-CCI patients [9].

A study from Japan correlated postoperative complications with worse survivals in gastric cancer patients. Patients with a CCI ≥ 32.15 had significantly lower 5-year overall and disease-specific survivals than those observed in the CCI low group. Moreover, a multivariate analysis identified the CCI as an independent prognostic indicator [25]. Another study from China similarly investigated the role of CCI in the setting of gastric cancer. Patients with high CCI (≥ 26.2) presented 5-year cancer-specific survival rates markedly inferior (46.3% vs. 54.9%) [26].

CCI was also correlated with several parameters of poor outcome after surgery, further explaining its potential role as a predictor of poor early outcomes. A study from Spain correlated CCI with the frailty status in elderly patients treated with surgery, suggesting a correlation among frailness, post-surgical complications, and poor outcomes [27]. Another US study showed a correlation between CCI and time to normal activity in patients undergoing gastrointestinal and hepato-bilio-pancreatic surgery [28].

Up to now, only one Dutch study has revealed a prognostic role of CCI in the specific setting of liver transplantation. Specifically, when transplants performed with organs from deceased-cardiac donors (DCD) or deceased-brain donors (DBD) were compared, 6-month postoperative median CCI was significantly higher in case of DCD grafts (53.4 vs 47.2). Moreover, more DCD recipients underwent re-transplantation for ischemic-type biliary lesions in this period (4% vs 1%), therefore suggesting a correlation between CCI and the development of biliary complications [11].

In the present experience, the CCI reported the best diagnostic ability respect to all the other tested scores in terms of graft loss risk, with AUCs of 0.94 and 0.77 in the training and validation sets for the diagnosis of 90-day graft loss. As for 1-year graft loss, CCI showed similar good performances in the training (AUC = 0.88) and validation sets (AUC = 0.75).

The strength of the CCI was particularly evident in light of the poor performances observed by the other tested pre- and post-transplant scores. Interestingly, no one of them ever showed an AUC > 0.70 in both the 90-day and 1-year graft loss risk estimation.

We also tested several CCI cut-offs. Interestingly, the value corresponding to the third quartile (47.3) was substantially similar to the threshold identified by Muller et al. on 7492 patients transplanted in 17 different centers [24].

We clearly understand that the diagnostic utility of CCI should appear marginal, mainly in consideration of the potentially long time required for its calculation. Typically, scores based on post-transplant data are collected within seven to ten days from LT [7, 23]. While the CCI calculation was set in our study at the time of patient discharge. However, just for clarifying the timeframes required for the CCI estimation, 1067/1262 (84.5%) and 464/520 (89.2%) patients were discharged in the training and validation sets within one month from LT, thus consenting to obtain the CCI calculation in an acceptable time, mainly in light of its usefulness for the prediction of 1-year graft loss.

Another aspect to consider is the fact that, once a LT patient has developed a complication, the ability to improve the patient outcomes should be markedly limited if compared with the possibility to pre-operatively prevent this specific complication. We understand this shortcoming of the model, obviously limiting the impact on the CCI in conditioning important aspects like an early re-transplantation. However, we think the role of the CCI merits consideration, mainly in light of the possibility to identify patients that are more “fragile” at the discharge time.

As an example, the sub-analysis focused on the transplants performed using aged grafts showed that the CCI even improved its diagnostic ability to predict early graft loss, therefore underlying the potential utility of this score in identifying transplanted patients at time of discharge requiring particular attentions during the follow-up.

As previously reported, the CCI should play a role in the prognosis of tumoral patients [10, 25, 26]. Studies on colorectal metastases and gastric cancer have been reported [10, 25, 26], while no studies have been published up to now with the intent to correlate CCI and post-transplant HCC recurrence risk. The present study was not constructed with the aim of investigating the correlation between HCC, LT and CCI. However, we can postulate that, also in this case, a worse correlation between high CCI and cancer should exist. Therefore, in tumor patients with high CCI at time of discharge, we should justify the use of a tailored immunosuppression (i.e., everolimus, rapid steroid withdrawal), a more cautious use of steroid boluses in the management of acute rejections, or a personalized scheme of outpatient follow-up (i.e., more frequent measurements of alpha-fetoprotein or a more stringent imaging protocol). Further studies specifically focused on the correlation between CCI and HCC are required.

Another important element is the potential correlation between CCI and biliary complications. Only one study specifically reported this connection, therefore requiring more detailed studies with the intent to clarify the potential intercorrelation between poor initial clinical course and biliary complications [9]. However, also in this case, we can postulate that the early identification of patients with a greater risk for biliary complications should offer the opportunity to design tailored therapies (i.e., ursodesoxycholic acid) and personalized schemes of outpatient follow-up comprehending early magnetic resonance imaging, with the intent to minimize possible complications.

We think such an opportunity is not of marginal relevance. As an example, 176/1262 (13.9%) and 102/520 (19.6%) patients in the training and validation sets overpassing the identified threshold of 47.3 were alive at discharging time. In the training set, 67/102 (38.1%) of these patients had biliary complications, and 40 (22.7%) required a re-transplantation during their follow-up. We are confident that these patients should potentially benefit from a modification of the post-discharge management policy, due to the peculiar condition derived from a complex post-transplant course.

One can argue that a potential bias of the study is represented by the arbitrary decision to calculate the CCI only at the time of the first post-LT hospitalization, excluding the possible complications observed by the patient after discharge. As an example, in the study by Muller et al., the CCI value was calculated at 12 months after transplantation [24]. On the opposite, we voluntarily decided to measure the CCI at the time of discharge. In fact, we think that such a measurement gives the opportunity to identify a sub-class of high-risk cases at discharge in which the previously reported management changes should be adopted with the intent to minimize their predictable poorer clinical course.

The intent of the study was not to compare the training and the validation sets. However, in light of the observed results, we noted that the Validation Set reported better CCI and 90-day results despite a higher median MELD value. We can do some suppositions for explaining this paradoxical result. First, MELD alone is not necessarily able to capture the overall recipient technical difficulties, mainly in case of “exception” pathologies like HCC and biliary cholangiopathies. In approximately half of the Training and Validation Set cases, we exactly observed these types of pathologies, respectively. Second, the high number of HCC cases in the training set should explain the higher rate of vascular thromboses/stenoses as a consequence of several intra-arterial treatments caused by bridging/downstaging strategies [29, 30]. Third, a possible effect of institutional case volume should explain this result. The Validation Set is, in fact, a high-volume center, representing a centralized referral and management center for all the hepatopathies of its country. Several studies already reported better results in high- vs. medium-volume centers [31, 32]. Another aspect to consider is the higher percentage of PNF observed in the training set (2.9 vs. 0.6%, *p* = 0.001), potentially explainable with worse histological aspects of the used graft. Unfortunately, due to the retrospective nature of the study, we were not able to explore this aspect. Last, we cannot exclude the presence of comorbidities like refractory ascites and portal hypertension in the recipients potentially justifying the observed results. Also in this case, we were not able to retrospectively evaluate in detail these aspects.

We are aware that the study may have some limitations. First, the study is retrospective and pluricentric. The main concern connected with the retrospective nature of the study is the risk of missed post-LT complications, mainly for the grade I–II cases. A systematic retrospective collection of all the pharmacological needs of LT patients should be challenging. However, we can say that, although potentially underestimated, the diagnostic effect of CCI was particularly relevant in our series. Consequently, we can only assert that the CCI role should be even stronger in a prospectively collected database.

Another limit of the study is connected with the fact that some complications have been classified for CCI scoring on a non-empirical basis, due to a lack of literature investigating on this aspect. Obviously, such a condition is of relevance because the heterogeneity in the CCI grading of specific complications may influence the performance characteristics of the score.

As for the multicentricity of the training set, we should emphasize that the large sample size of this population should minimize possible biases related to data analysis. Moreover, the validation set was based only on a monocentric experience, however confirming an overall broad prognostic ability of CCI also in this context.

In conclusion, the CCI shows a very good diagnostic ability for 90-day and 1-year graft loss in both a multicentric training and a monocentric validation set. Its diagnostic power is superior to other commonly adopted pre- and post-LT scores. Further analyses are required to prove its validity even in the long term.

## Abbreviations

AUC: Area under the curve
BAR: Balance of risk
CCI: Comprehensive complication index
CI: Confidence intervals
DBD: Deceased-brain donor
DCD: Deceased-cardiac donor
DOR: Diagnostic odds ratio
EAD: Early allograft dysfunction
HR: Hazard ratio
IQR: Interquartile ranges
LDLT: Living donor
LT: Liver transplantation
MELD: Model for end-stage liver disease
MOF: Multiorgan failure
PNF: Primary non-function
RRT: Renal replacement therapy
SOFT: Survival outcomes following liver transplantation

## References

[1] Kamath PS, Wiesner RH, Malinchoc M (2001). A model to predict survival in patients with end-stage liver disease. Hepatology.

[2] Freeman RB (2012). A decade of model for end-stage liver disease: lessons learned and need for re-evaluation of allocation policies. Curr Opin Organ Transplant.

[3] Silberhumer GR, Hetz H, Rasoul-Rockenschaub S (2006). Is MELD score sufficient to predict not only death on waiting list, but also post-transplant survival?. Transplant Int.

[4] Klein KB, Stafinski TD, Menon D (2013). Predicting survival after liver transplantation based on pre-transplant MELD score: a systematic review of the literature. PLoS ONE.

[5] Halldorson JB, Bakthavatsalam R, Fix O (2009). D-MELD, a simple predictor of post liver transplant mortality for optimization of donor/recipient matching. Am J Transplant.

[6] Dutkowski P, Oberkofler CE, Slankamenac K (2011). Are there better guidelines for allocation in liver transplantation? A novel score targeting justice and utility in the model for end-stage liver disease era. Ann Surg.

[7] Olthoff KM, Kulik L, Samstein B (2010). Validation of a current definition of early allograft dysfunction in liver transplant recipients and analysis of risk factors. Liver Transplant.

[8] Slankamenac K, Graf R, Barkun J (2013). The comprehensive complication index: a novel continuous scale to measure surgical morbidity. Ann Surg.

[9] Yamashita S, Sheth RA, Niekamp AS (2017). Comprehensive complication index predicts cancer-specific survival after resection of colorectal metastases independent of RAS mutational status. Ann Surg.

[10] Rössler F, Sapisochin G, Song G (2016). Defining benchmarks for major liver surgery: a multicentre analysis of 5202 living liver donors. Ann Surg.

[11] Kalisvaart M, de Haan JE, Polak WG (2017). Comparison of postoperative outcomes between donation after circulatory death and donation after brain death liver transplantation using the comprehensive complication index. Ann Surg.

[12] Dindo D, Demartines N, Clavien PA (2004). Classification of surgical complications: a new proposal with evaluation in a cohort of 6336 patients and results of a survey. Ann Surg.

[13] Sainani KL (2013). Multivariate regression: the pitfalls of automated variable selection. PM R.

[14] Freeman RB, Wiesner RH, Harper A, UNOS/OPTN Liver Disease Severity Score, UNOS/OPTN Liver and Intestine, and UNOS/OPTN Pediatric Transplantation Committees (2002). The new liver allocation system: moving toward evidence-based transplantation policy. Liver Transplant.

[15] Duan BW, Lu SC, Wu JS (2014). Model for end-stage liver disease (MELD) score does not predict outcomes of hepatitis B-induced acute-on-chronic liver failure in transplant recipients. Transplant Proc.

[16] Yadav SK, Saraf N, Saigal S (2017). High MELD score does not adversely affect outcome of living donor liver transplantation: experience in 1000 recipients. Clin Transplant.

[17] Weismüller TJ, Fikatas P, Schmidt J (2011). Multicentric evaluation of model for end-stage liver disease-based allocation and survival after liver transplantation in Germany—limitations of the ‘sickest first’-concept. Transplant Int.

[18] Rana A, Hardy MA, Halazun KJ (2008). Survival outcomes following liver transplantation (SOFT) score: a novel method to predict patient survival following liver transplantation. Am J Transplant.

[19] Montano-Loza AJ, Duarte-Rojo A, Meza-Junco J (2015). Inclusion of sarcopenia within MELD (MELD-Sarcopenia) and the prediction of mortality in patients with cirrhosis. Clin Transl Gastroenterol.

[20] Avolio AW, Cillo U, Salizzoni M, Donor-to-Recipient Italian Liver Transplant (D2R-ILTx) Study Group (2011). Balancing donor and recipient risk factors in liver transplantation: the value of D-MELD with particular reference to HCV recipients. Am J Transplant.

[21] Ma Y, Wang Q, Yang J (2015). Comparison of different scoring systems based on both donor and recipient characteristics for predicting outcome after living donor liver transplantation. PLoS ONE.

[22] de Campos Junior ID, Stucchi RS, Udo EY (2015). Application of the BAR score as a predictor of short- and long-term survival in liver transplantation patients. Hepatol Int.

[23] Pareja E, Cortes M, Hervás D (2015). A score model for the continuous grading of early allograft dysfunction severity. Liver Transplant.

[24] Muller X, Marcon F, Sapisochin G (2018). Defining benchmarks in liver transplantation: a multicentre outcome analysis determining best achievable results. Ann Surg.

[25] Shimizu S, Saito H, Kono Y (2019). The prognostic significance of the comprehensive complication index in patients with gastric cancer. Surg Today.

[26] Tu RH, Lin JX, Li P (2018). Comprehensive complication index predicts cancer-specific survival of patients with postoperative complications after curative resection of gastric cancer. Gastroenterol Res Pract.

[27] Artiles-Armas M, Roque-Castellano C, Conde-Martel A, Marchena-Gómez J (2019). The comprehensive complication index is related to frailty in elderly surgical patients. J Surg Res.

[28] Ray S, Mehta NN, Mangla V (2019). A comparison between the comprehensive complication index and the Clavien-Dindo grading as a measure of postoperative outcome in patients undergoing gastrointestinal surgery—a prospective study. J Surg Res.

[29] Goel A, Mehta N, Guy J (2014). Hepatic artery and biliary complications in liver transplant recipients undergoing pretransplant transarterial chemoembolization. Liver Transplant.

[30] Sneiders D, Houwen T, Pengel LHM (2018). Systematic review and meta-analysis of posttransplant hepatic artery and biliary complications in patients treated with transarterial chemoembolization before liver transplantation. Transplantation.

[31] Yoo S, Jang EJ, Yi NJ (2019). Effect of institutional case volume on in-hospital mortality after living donor liver transplantation: analysis of 7073 cases between 2007 and 2016 in Korea. Transplantation.

[32] Ozhathil DK, Li YF, Smith JK (2011). Impact of center volume on outcomes of increased-risk liver transplants. Liver Transplant.

